# Supplementary material for: Knowledge-informed multimodal cfDNA analysis improves sensitivity and generalization in cancer detection
Source: bioRxiv. 2025 Oct 21:2025.10.20.683167. Preprint. [Version 1] doi: 10.1101/2025.10.20.683167 (PMC12633305; doi:10.1101/2025.10.20.683167)
Supplement: 1 [file NIHPP2025.10.20.683167V1-supplement-1.pdf]

bioRxiv preprint doi: https://doi.org/10.1101/2025.10.20.683167; this version posted October 21, 2025. The copyright holder for this preprint (which was not certified by peer review) is the author/funder, who has granted bioRxiv a license to display the preprint in perpetuity. It is made available under aCC-BY 4.0 International license.

Supplementary Figure Legends

Figure S1. Technical variations effect on Fragment Length Distribution and the distribution of features in early-stage. A, Boxplots show the distribution of the tumor fraction (TF) estimated by IchorCNA[36] across multiple studies. The dotted lines show the mean tumor fraction of healthy samples (green) and tumor samples (red) in each study. **B,** Density of cfDNA fragment lengths observed in cancer samples across multiple studies. **C,** Boxplots show the distribution of Fate-AI features (Methods) in healthy controls and early-stage cancer samples with low TF less than 0.03. Statistical comparisons were performed using the Wilcoxon rank-sum test (ns: p-value > 0.05, * p-value ≤ 0.05, ** p-value ≤ 0.01, *** p-value ≤ 0.001, **** p-value $<= 0.0001$). A,C: Boxplots show the median as center, the lower and upper hinges that correspond to the 25th and the 75th percentile, and whiskers that extend to the smallest and largest value no more than 1.5*IQR. Values that stray more than 1.5*IQR upwards or downwards from the whiskers are considered potential outliers and represented with dots.

Figure S2. Data Cohort. Sankey diagram showing the composition of the study cohorts. Samples are stratified by class (left), study of origin (middle-left), internal data (brown), external data (light blue), sample type (middle-right), and sequencing-based approaches (right). The diagram illustrates the samples across categories, including pre-treatment, post-treatment, and control groups, as well as low-pass whole-genome sequencing (LPWGS) and LPWGS combined with cfMeDIP-seq data. Sample counts for each category are indicated in parentheses.

Figure S3. Performance evaluation of methods for cancer detection in LPWGS-cfMeDIP-seq. A, ROC curves illustrating classification performance in matched-cohort setting for melanoma (melanoma n=14, healthy n=61), PM (PM n=20, healthy n=61), and BLCA (BLCA n=38, healthy n=61), data from this study. **B,** Heatmap summarizing the sensitivity achieved by multiple methods in the various settings: matched-cohort on data from this study, matched-cohort on Baca et al. [25], cross-cohort on Baca et al. [25] with trained model on this study. **C,** Same as in B, for the specificity. **D,** ROC curves illustrating classification performance in matched-cohort setting for BC (BC n=15, healthy n=33), CRC (CRC n=29, healthy n=33), lung cancer (lung cancer n=38, healthy n=33), and PRAD (PRAD n=28, healthy n=33) from Baca et. al [25]. **E,** Heatmap of feature importance across different cancer types for the Fate-AI (left) and Fate-AI (+Meth) (right). Features are sorted by mean importance across studies, settings, and cancer types. Color intensity represents feature importance from low (white) to high (blue). Features are grouped into fragmentomics and methylation categories.

bioRxiv preprint doi: https://doi.org/10.1101/2025.10.20.683167; this version posted October 21, 2025. The copyright holder for this preprint (which was not certified by peer review) is the author/funder, who has granted bioRxiv a license to display the preprint in perpetuity. It is made available under aCC-BY 4.0 International license.

Figure S4. Performance evaluation of methods for cancer detection in LPWGS. **A,** ROC curves illustrating classification performance (melanoma vs healthy) when the model is trained on data from this study and Baca et. al 2023 (melanoma n=20, healthy n=94) and tested on the dataset from Widman et al. 2024 study (melanoma n=38, healthy n=35). **B,** Boxplots of predicted scores stratified by healthy (H) and cancer stage (III-IV) for melanoma (n=38). **C,** ROC curves illustrating classification performance (PDAC vs healthy) for the model trained with cross-validation with data from this study (PDAC n=49, healthy n=69). **D,** Boxplots of predicted scores stratified by healthy (H) and cancer stage (I–IV) for PDAC (n=49). **E,** ROC curves illustrating classification performance (CRC vs healthy) when the model is trained on data from this study and tested on the dataset from Hallermayr et al. study [35] (CRC n=43, healthy n=61). **F,** Boxplots of predicted scores stratified by healthy (H) and cancer stage (I–IV) for CRC (n=43).
B,D,F: Boxplots represent the interquartile range (IQR), horizontal lines mark medians, and whiskers denote data points within 1.5×IQR. Significance was computed by a two−sided Wilcoxon rank-sum test (ns: p-value > 0.05, * p-value ≤ 0.05, ** p-value ≤ 0.01, *** p-value ≤ 0.001, **** p-value $<= 0.0001$).

Figure S5. Longitudinal monitoring of colon rectal carcinoma patients. Longitudinal scores from multiple classification methods (Fate-AI, Delfi, Griffin, IchorCNA) for CRC patients (LB-CRC:1, 7, 8, 15, 20, 21, 29, 35, 38, 42, 43, 46, 51, 47, 48, 52, 57, 59) [35]. Each panel shows score dynamics over time, with therapy (red lines) and staging events (blue lines) annotated.

Figure S6. Longitudinal monitoring of multiple myeloma patients. **A,** The model was applied to 91 longitudinal samples at up to four time points (average four samples per patient, range 2-4) from 24 MM cases. Patients are stratified based on progression status: no progression (n=12) and progression (n=12). The x axis represents the day relative to maintenance start date and the y axis represents the score of Fate-AI (fragmentomic features only). Each panel shows score dynamics over time for each patient with progression event (red lines) and disease measurements that include: Monoclonal protein (M_pro), Light Chain (Light C), MRD status, and Positron Emission Tomography (PET).

Figure S7. Temporal evaluation in patients before and after treatment. **A,** Post-treatment Fate-AI scores over time stratified by relapse and remission status, in the post-treatment samples of ES cohort [21]; points indicate group means and error bars represent variance. **B,** Boxplots showing Fate-AI scores (matched-cohort) between patients who experienced a relapse event and those who remained relapse-free, in the pre-treatment samples of ES cohort [21]. **C,** Scores from multiple classification methods (Fate-AI, Delfi, Griffin, IchorCNA) for pre-treatment samples of PM patients. Boxplots showing scores for each method (matched-cohort), contrasting patients with a treatment response. **D,** Boxplots showing Fate-AI scores (cross-cohort setting, training on Baca et al. [25] and this study) in post-treatment samples of the conventional immunotherapy Melanoma cohort [15]. Left panel: Fate-AI scores among BOR response categories: complete response (CR), partial response (PR), stable disease (SD), and progression disease (PD). Right panel: Fate-AI scores between patients who experienced a progression event and those who remained progression-free.
B,C,D: Boxplots show the median as the center, the lower and upper hinges that correspond to the 25th and the 75th percentile, and whiskers that extend to the smallest and largest value no more than

bioRxiv preprint doi: https://doi.org/10.1101/2025.10.20.683167; this version posted October 21, 2025. The copyright holder for this preprint (which was not certified by peer review) is the author/funder, who has granted bioRxiv a license to display the preprint in perpetuity. It is made available under aCC-BY 4.0 International license.

1.5*IQR. Values that stray more than 1.5*IQR upwards or downwards from the whiskers are considered potential outliers and represented with dots. A,B,C,D: Significance was computed by a two−sided Wilcoxon rank-sum test (ns: p-value > 0.05, * p-value ≤ 0.05, ** p-value ≤ 0.01, *** p-value ≤ 0.001, **** p-value $<= 0.0001$).

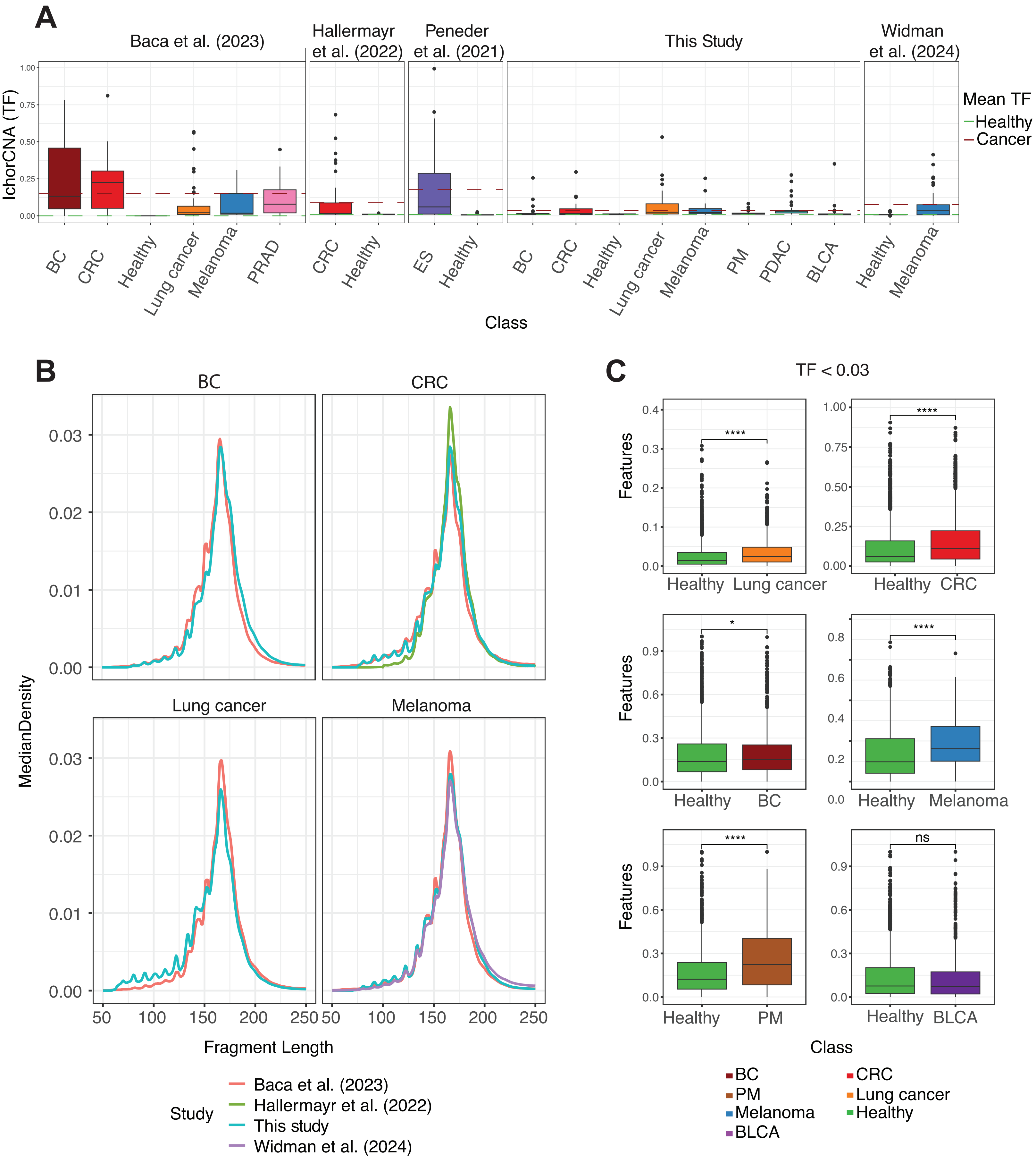

A
Baca et al. (2023)
Hallermayr et al. (2022)
Peneder et al. (2021)
This Study
Widman et al. (2024)
IchorCNA (TF)
Mean TF
Healthy
Cancer
BC
CRC
Healthy
Lung cancer
Melanoma
PRAD
ES
PM
PDAC
BLCA
Class
B
MedianDensity
Fragment Length
Study
Baca et al. (2023)
Hallermayr et al. (2022)
This study
Widman et al. (2024)
C
TF < 0.03
Features
Class

Figure S1

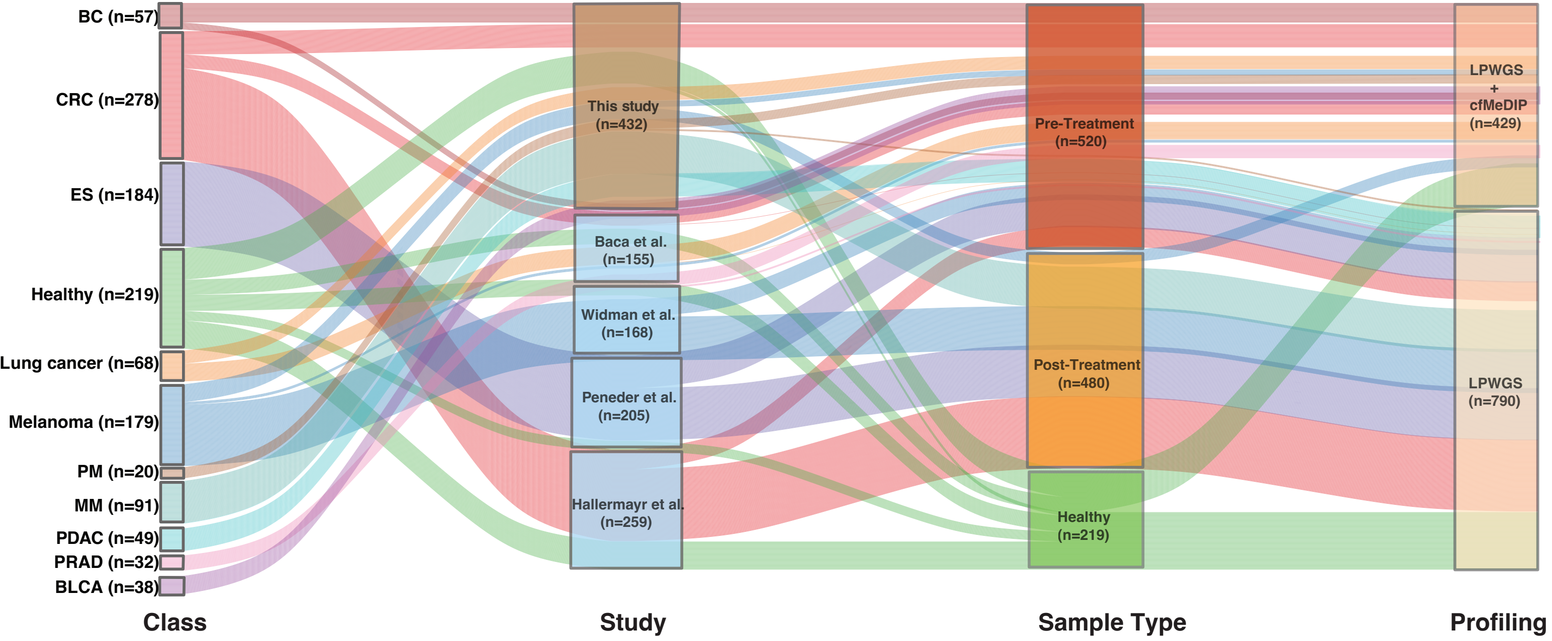
BC (n=57)
CRC (n=278)
ES (n=184)
Healthy (n=219)
Lung cancer (n=68)
Melanoma (n=179)
PM (n=20)
MM (n=91)
PDAC (n=49)
PRAD (n=32)
BLCA (n=38)
This study (n=432)
Baca et al. (n=155)
Widman et al. (n=168)
Peneder et al. (n=205)
Hallermayr et al. (n=259)
Pre-Treatment (n=520)
Post-Treatment (n=480)
Healthy (n=219)
LPWGS + cfMeDIP (n=429)
LPWGS (n=790)
Class
Study
Sample Type
Profiling

Figure S2

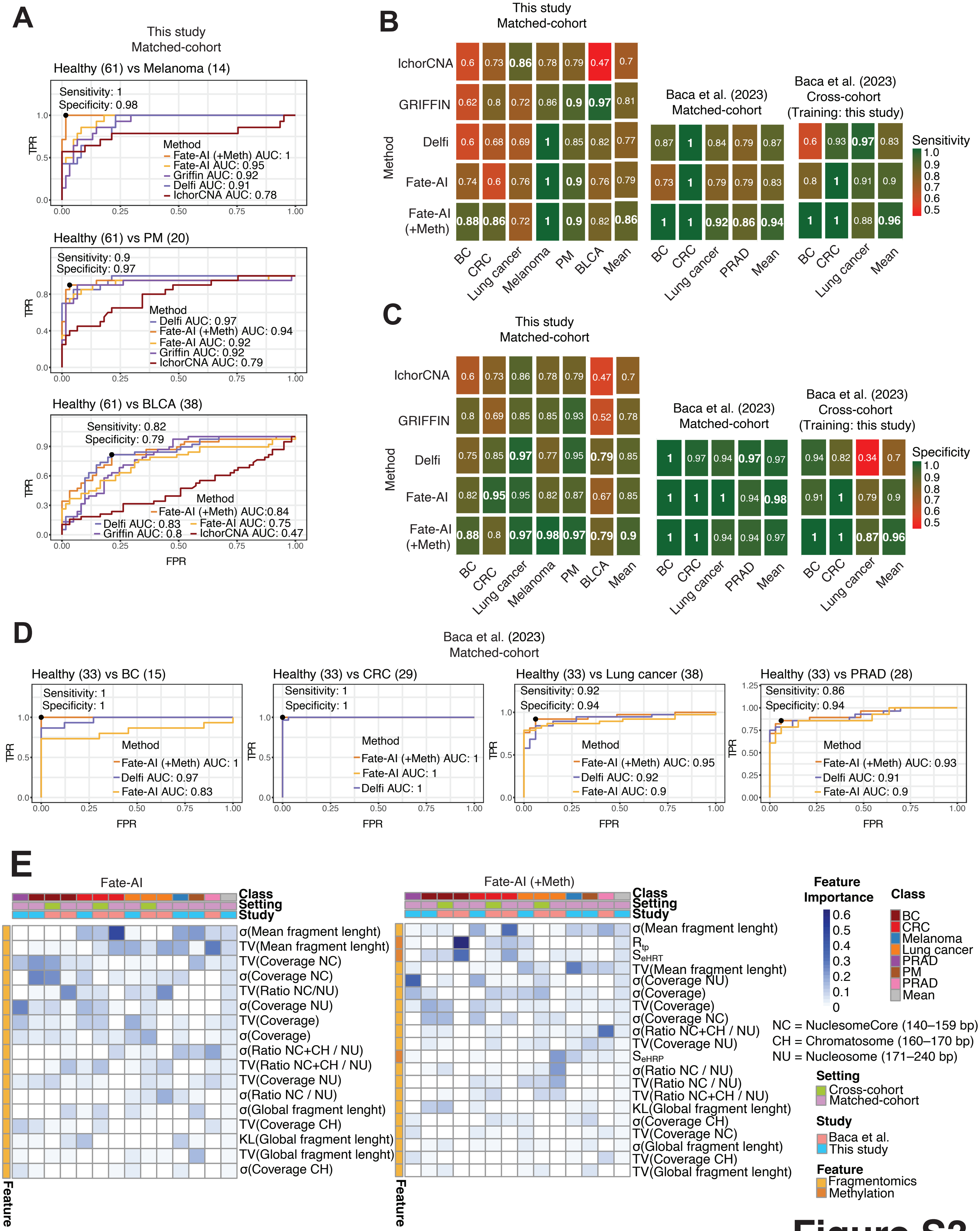
A
This study
Matched-cohort
Healthy (61) vs Melanoma (14)
Sensitivity: 1
Specificity: 0.98
Method
Fate-AI (+Meth) AUC: 1
Fate-AI AUC: 0.95
Griffin AUC: 0.92
Delfi AUC: 0.91
IchorCNA AUC: 0.78
Healthy (61) vs PM (20)
Sensitivity: 0.9
Specificity: 0.97
Method
Delfi AUC: 0.97
Fate-AI (+Meth) AUC: 0.94
Fate-AI AUC: 0.92
Griffin AUC: 0.92
IchorCNA AUC: 0.79
Healthy (61) vs BLCA (38)
Sensitivity: 0.82
Specificity: 0.79
Method
Fate-AI (+Meth) AUC:0.84
Delfi AUC: 0.83
Fate-AI AUC: 0.75
Griffin AUC: 0.8
IchorCNA AUC: 0.47
TPR
FPR
B
This study
Matched-cohort
Baca et al. (2023)
Matched-cohort
Baca et al. (2023)
Cross-cohort
(Training: this study)
Method
IchorCNA
GRIFFIN
Delfi
Fate-AI
Fate-AI (+Meth)
BC
CRC
Lung cancer
Melanoma
PM
BLCA
PRAD
Mean
Sensitivity
C
Specificity
D
Baca et al. (2023)
Matched-cohort
Healthy (33) vs BC (15)
Sensitivity: 1
Specificity: 1
Method
Fate-AI (+Meth) AUC: 1
Delfi AUC: 0.97
Fate-AI AUC: 0.83
Healthy (33) vs CRC (29)
Sensitivity: 1
Specificity: 1
Method
Fate-AI (+Meth) AUC: 1
Fate-AI AUC: 1
Delfi AUC: 1
Healthy (33) vs Lung cancer (38)
Sensitivity: 0.92
Specificity: 0.94
Method
Fate-AI (+Meth) AUC: 0.95
Delfi AUC: 0.92
Fate-AI AUC: 0.9
Healthy (33) vs PRAD (28)
Sensitivity: 0.86
Specificity: 0.94
Method
Fate-AI (+Meth) AUC: 0.93
Delfi AUC: 0.91
Fate-AI AUC: 0.9
E
Fate-AI
Fate-AI (+Meth)
Class
Setting
Study
Feature
σ(Mean fragment lenght)
TV(Mean fragment lenght)
TV(Coverage NC)
σ(Coverage NC)
TV(Ratio NC/NU)
σ(Coverage NU)
TV(Coverage)
σ(Coverage)
σ(Ratio NC+CH / NU)
TV(Ratio NC+CH / NU)
TV(Coverage NU)
σ(Ratio NC / NU)
σ(Global fragment lenght)
TV(Coverage CH)
KL(Global fragment lenght)
TV(Global fragment lenght)
σ(Coverage CH)
R_tp
S_eHRT
S_eHRP
TV(Ratio NC / NU)
Feature Importance
NC = NucleosomeCore (140–159 bp)
CH = Chromatosome (160–170 bp)
NU = Nucleosome (171–240 bp)
Class
BC
CRC
Melanoma
Lung cancer
PRAD
PM
PRAD
Mean
Setting
Cross-cohort
Matched-cohort
Study
Baca et al.
This study
Feature
Fragmentomics
Methylation

Figure S3

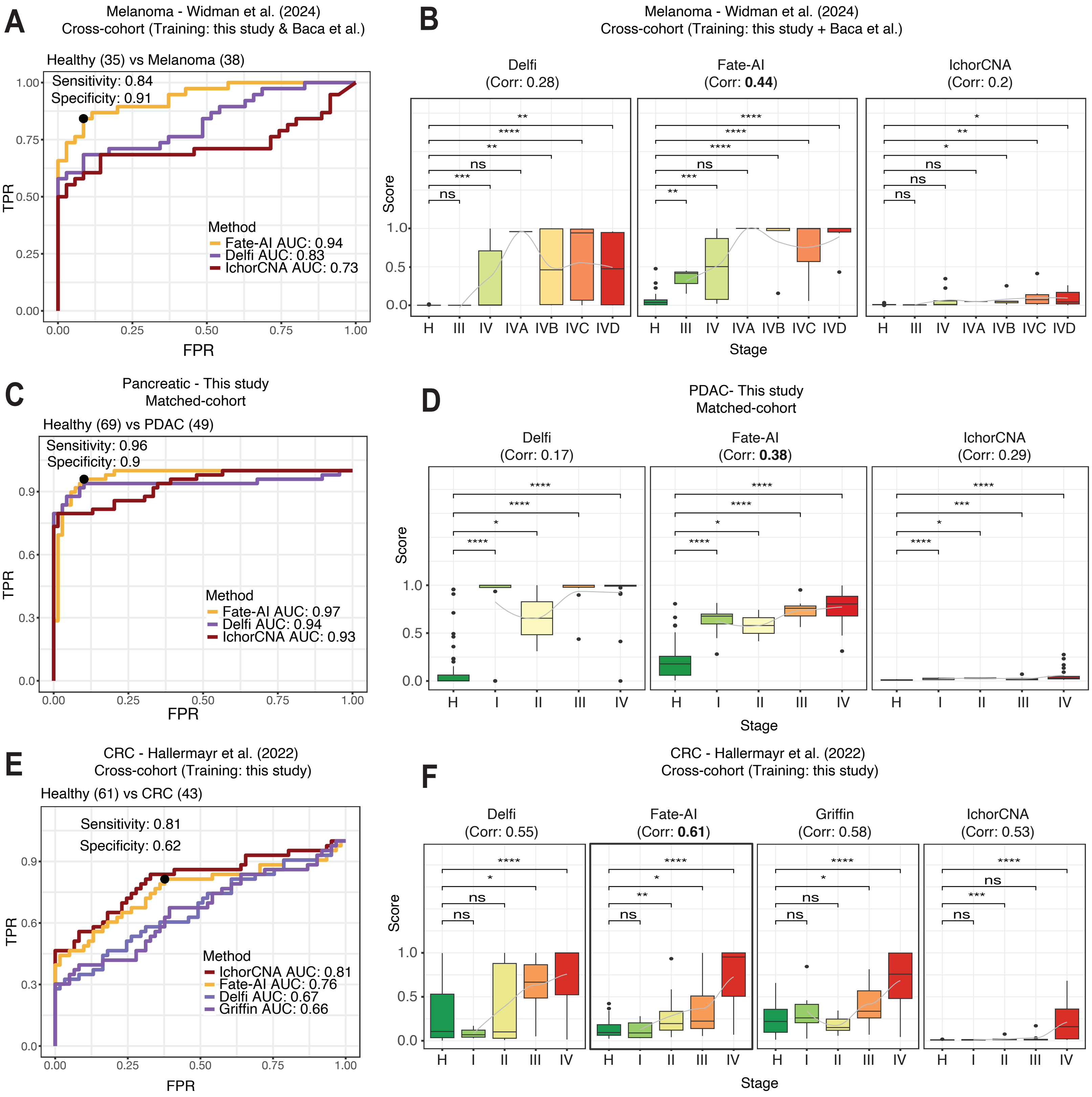
A
Melanoma - Widman et al. (2024)
Cross-cohort (Training: this study & Baca et al.)
Healthy (35) vs Melanoma (38)
Sensitivity: 0.84
Specificity: 0.91
Method
Fate-AI AUC: 0.94
Delfi AUC: 0.83
IchorCNA AUC: 0.73
TPR
FPR
B
Melanoma - Widman et al. (2024)
Cross-cohort (Training: this study + Baca et al.)
Delfi
(Corr: 0.28)
Fate-AI
(Corr: 0.44)
IchorCNA
(Corr: 0.2)
Score
Stage
H III IV IVA IVB IVC IVD
C
Pancreatic - This study
Matched-cohort
Healthy (69) vs PDAC (49)
Sensitivity: 0.96
Specificity: 0.9
Method
Fate-AI AUC: 0.97
Delfi AUC: 0.94
IchorCNA AUC: 0.93
TPR
FPR
D
PDAC- This study
Matched-cohort
Delfi
(Corr: 0.17)
Fate-AI
(Corr: 0.38)
IchorCNA
(Corr: 0.29)
Score
Stage
H I II III IV
E
CRC - Hallermayr et al. (2022)
Cross-cohort (Training: this study)
Healthy (61) vs CRC (43)
Sensitivity: 0.81
Specificity: 0.62
Method
IchorCNA AUC: 0.81
Fate-AI AUC: 0.76
Delfi AUC: 0.67
Griffin AUC: 0.66
TPR
FPR
F
CRC - Hallermayr et al. (2022)
Cross-cohort (Training: this study)
Delfi
(Corr: 0.55)
Fate-AI
(Corr: 0.61)
Griffin
(Corr: 0.58)
IchorCNA
(Corr: 0.53)
Score
Stage
H I II III IV

Figure S4

bioRxiv preprint doi: https://doi.org/10.1101/2025.10.20.683167; this version posted October 21, 2025. The copyright holder for this preprint (which was not certified by peer review) is the author/funder, who has granted bioRxiv a license to display the preprint in perpetuity. It is made available under aCC-BY 4.0 International license.

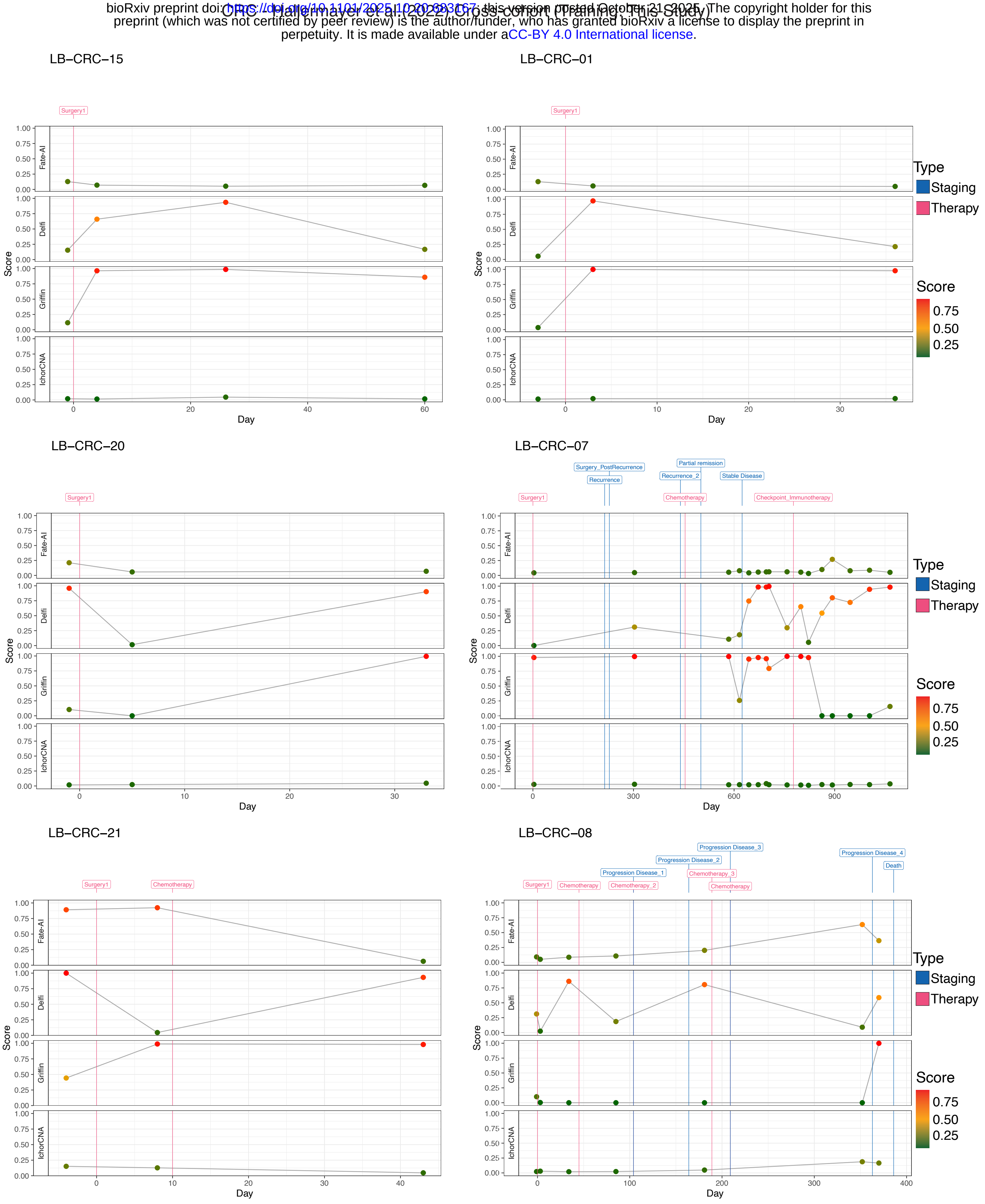

Figure S5

bioRxiv preprint doi: https://doi.org/10.1101/2025.10.20.683227; this version posted October 21, 2025. The copyright holder for this preprint (which was not certified by peer review) is the author/funder, who has granted bioRxiv a license to display the preprint in perpetuity. It is made available under aCC-BY 4.0 International license.

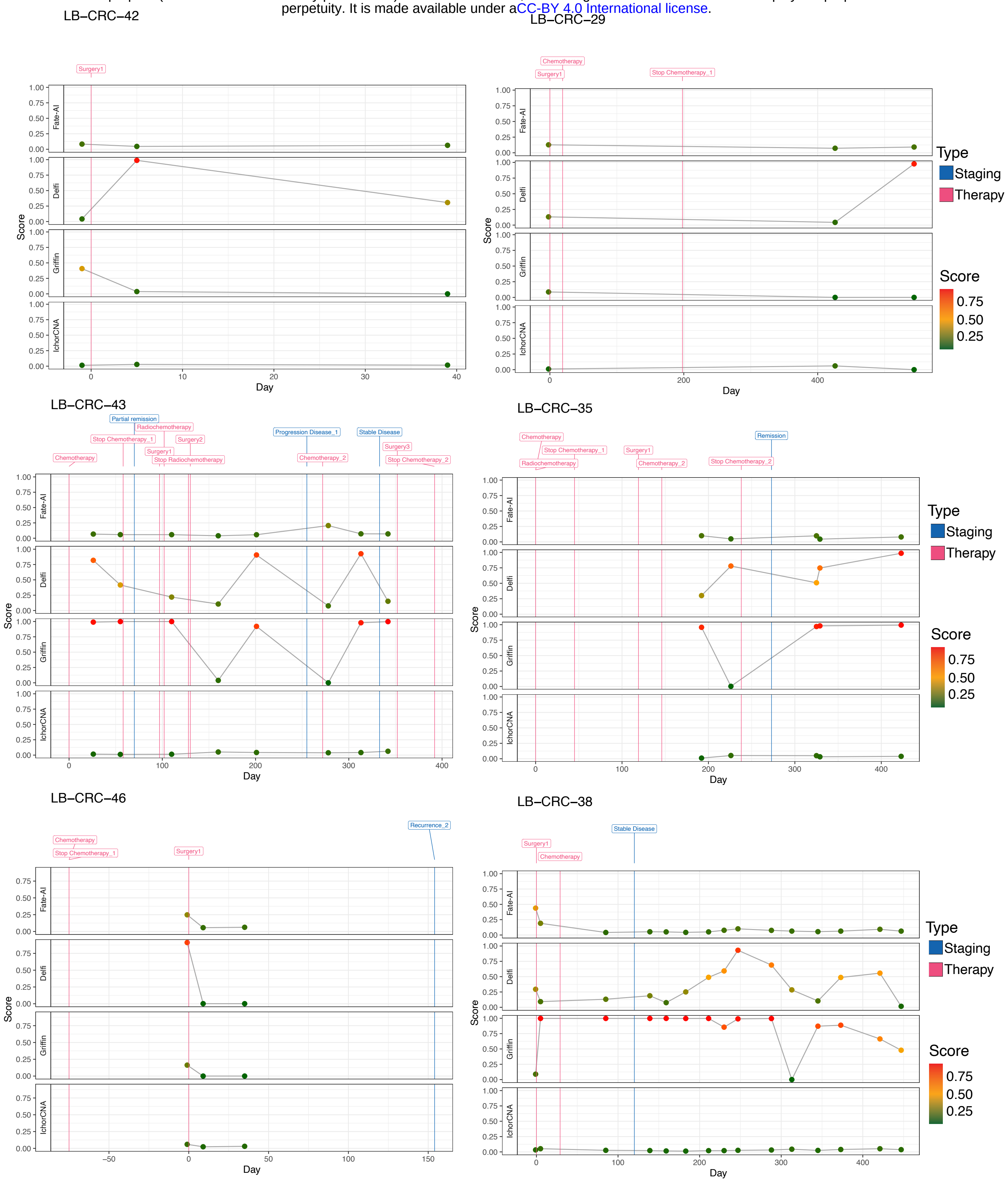

Figure S5 (continued)

bioRxiv preprint doi: https://doi.org/10.1101/2025.10.20.683167; this version posted October 21, 2025. The copyright holder for this preprint (which was not certified by peer review) is the author/funder, who has granted bioRxiv a license to display the preprint in perpetuity. It is made available under aCC-BY 4.0 International license.

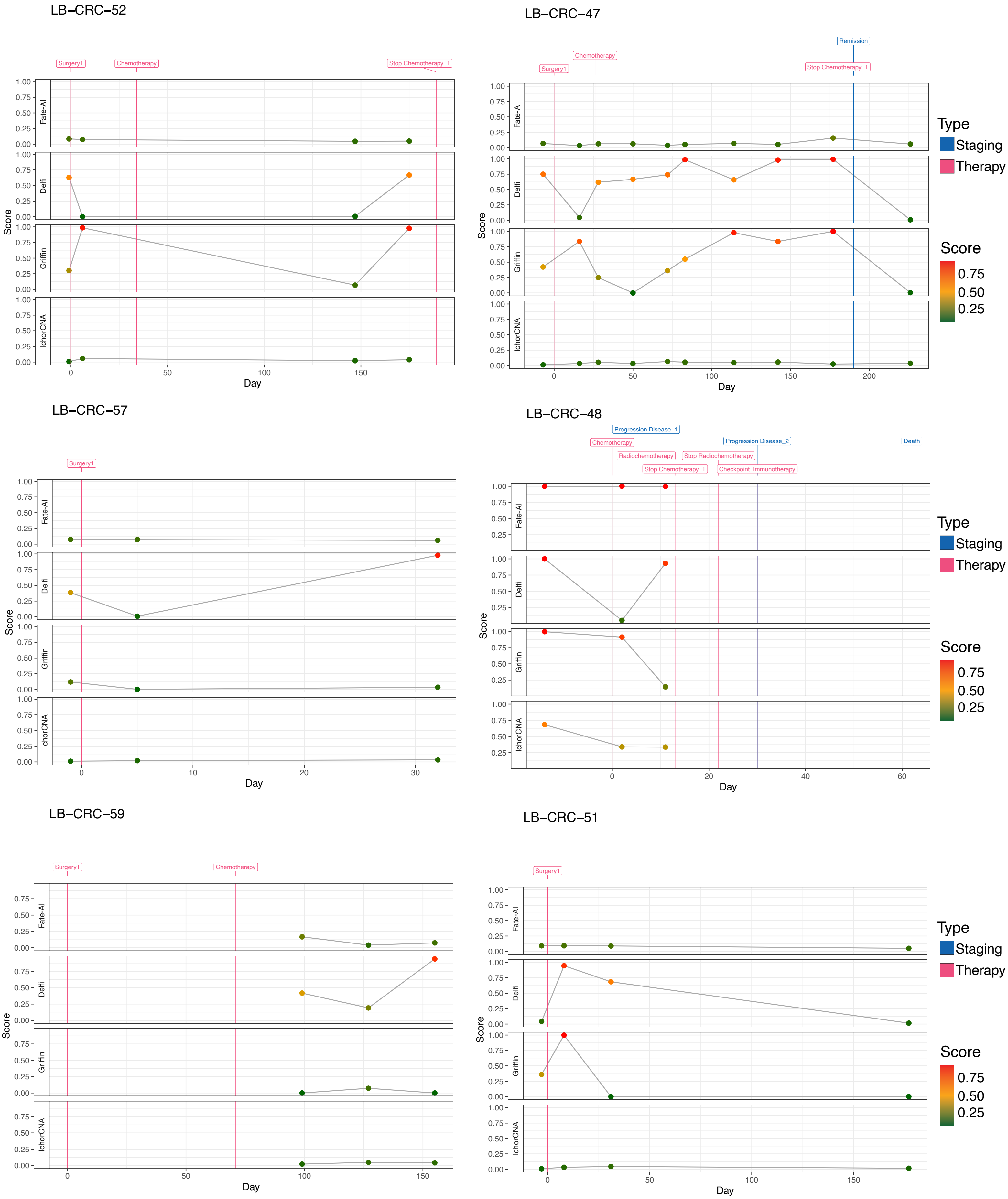

Figure S5 (continued)

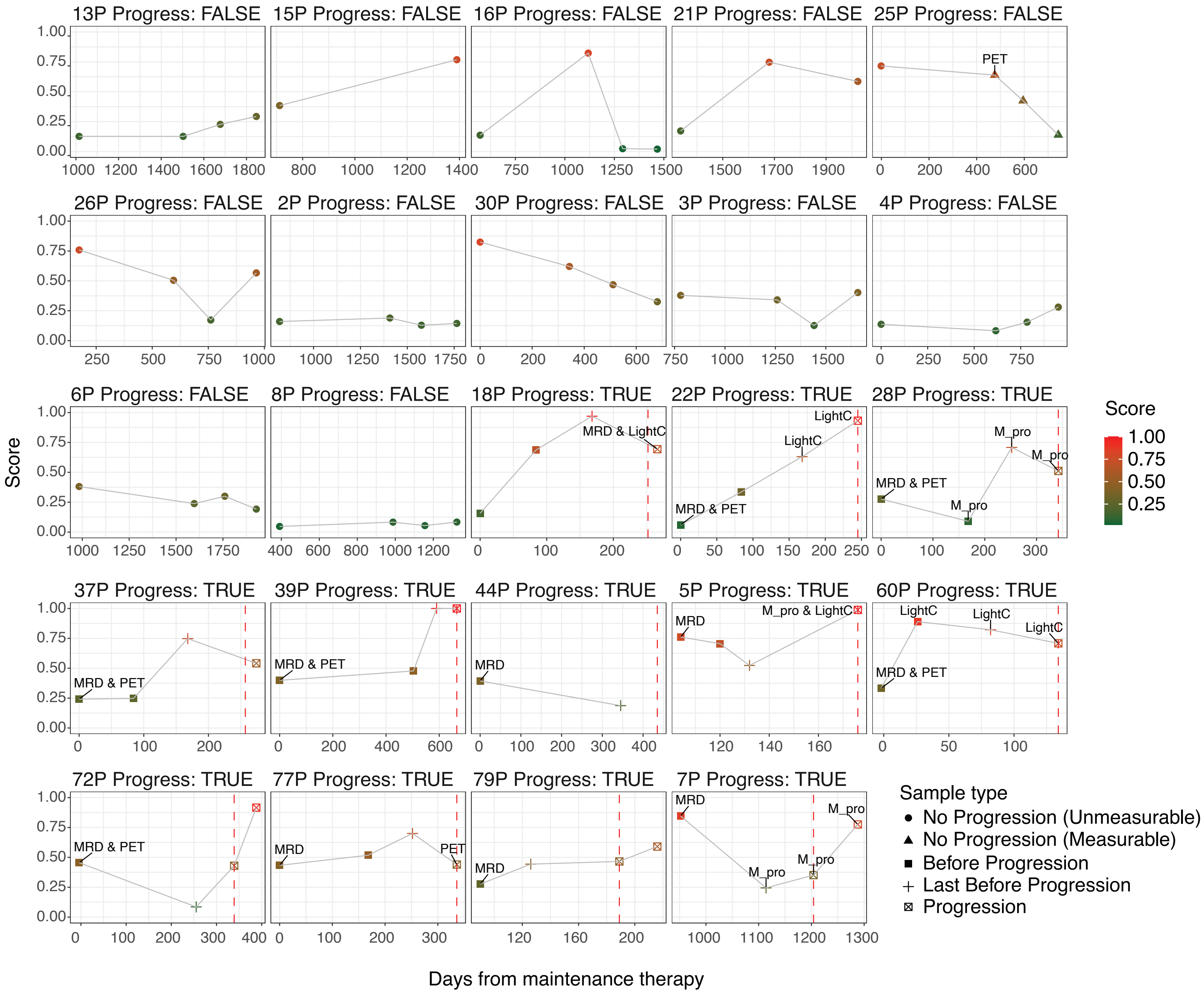

13P Progress: FALSE
15P Progress: FALSE
16P Progress: FALSE
21P Progress: FALSE
25P Progress: FALSE
26P Progress: FALSE
2P Progress: FALSE
30P Progress: FALSE
3P Progress: FALSE
4P Progress: FALSE
6P Progress: FALSE
8P Progress: FALSE
18P Progress: TRUE
22P Progress: TRUE
28P Progress: TRUE
37P Progress: TRUE
39P Progress: TRUE
44P Progress: TRUE
5P Progress: TRUE
60P Progress: TRUE
72P Progress: TRUE
77P Progress: TRUE
79P Progress: TRUE
7P Progress: TRUE
PET
MRD & LightC
LightC
MRD & PET
M_pro
MRD
M_pro & LightC
Score
Days from maintenance therapy
Score
1.00
0.75
0.50
0.25
Sample type
No Progression (Unmeasurable)
No Progression (Measurable)
Before Progression
Last Before Progression
Progression

Figure S6

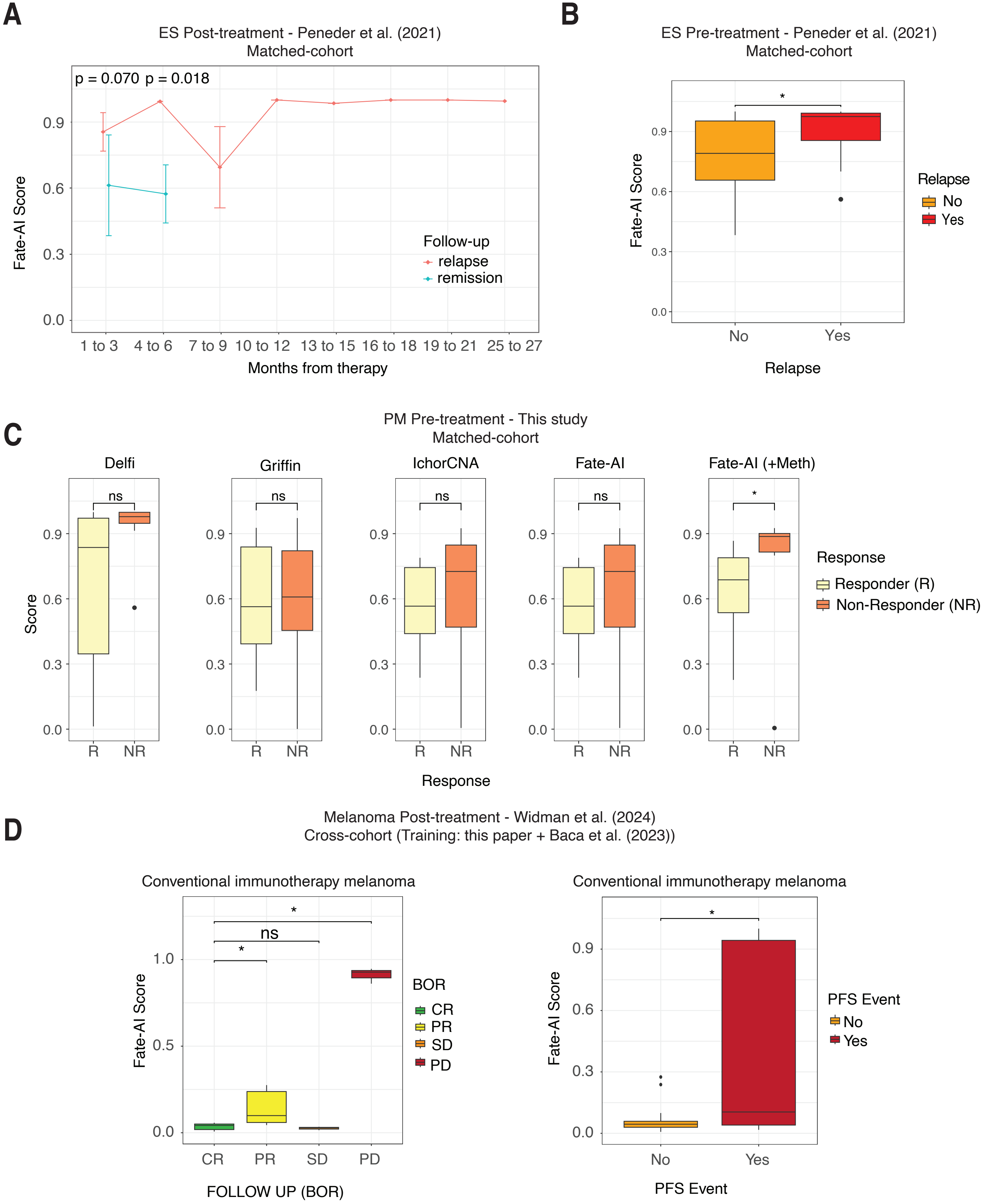
A
ES Post-treatment - Peneder et al. (2021)
Matched-cohort
p = 0.070 p = 0.018
Fate-AI Score
0.0
0.3
0.6
0.9
1 to 3
4 to 6
7 to 9
10 to 12
13 to 15
16 to 18
19 to 21
25 to 27
Months from therapy
Follow-up
relapse
remission
B
ES Pre-treatment - Peneder et al. (2021)
Matched-cohort
*
Fate-AI Score
0.0
0.3
0.6
0.9
No
Yes
Relapse
Relapse
No
Yes
C
PM Pre-treatment - This study
Matched-cohort
Delfi
Griffin
IchorCNA
Fate-AI
Fate-AI (+Meth)
ns
ns
ns
ns
*
Score
0.0
0.3
0.6
0.9
R
NR
Response
Response
Responder (R)
Non-Responder (NR)
D
Melanoma Post-treatment - Widman et al. (2024)
Cross-cohort (Training: this paper + Baca et al. (2023))
Conventional immunotherapy melanoma
*
ns
*
Fate-AI Score
0.0
0.5
1.0
CR
PR
SD
PD
FOLLOW UP (BOR)
BOR
CR
PR
SD
PD
Conventional immunotherapy melanoma
*
Fate-AI Score
0.0
0.3
0.6
0.9
No
Yes
PFS Event
PFS Event
No
Yes

Figure S7
